# Lactococcin B Is Inactivated by Intrinsic Proteinase PrtP Digestion in *Lactococcus lactis* subsp. *lactis* BGMN1-501

**DOI:** 10.3389/fmicb.2019.00874

**Published:** 2019-04-24

**Authors:** Goran Vukotic, Natalija Polovic, Nemanja Mirkovic, Branko Jovcic, Nemanja Stanisavljevic, Djordje Fira, Milan Kojic

**Affiliations:** ^1^Faculty of Biology, University of Belgrade, Belgrade, Serbia; ^2^Institute of Molecular Genetics and Genetic Engineering, University of Belgrade, Belgrade, Serbia; ^3^Faculty of Chemistry, University of Belgrade, Belgrade, Serbia

**Keywords:** proteinase PrtP, bacteriocin LcnB, hydrolysis, inactivation, *Lactococcus lactis*

## Abstract

In our previous study we demonstrated that proteinase PrtP is able to impair bacteriocin LcnB activity, despite being produced by the same organism and encoded by the same plasmid. However, precise mechanism of this action, i.e., the exact cleavage site within LcnB bacteriocin, as well as its effect on antimicrobial activity of the resulting peptide remained vague. Here we further explored the interplay between these two proteins and defined, using mass spectrometry, that this unusual hydrolysis indeed occurs *in vivo*, between the sixth and seventh amino acid on the N terminus of LcnB. To address whether the cleaved form of LcnB retains any level of activity, both recombinant and chemically synthesized variant of truncated LcnB were engineered and produced, but demonstrated no antimicrobial activity. When LcnB was recombinantly overexpressed and subjected to PrtP digestion, the change in its antimicrobial activity was monitored and the degradation products analyzed with reverse-phase high-pressure liquid chromatography. The results confirmed the inactivity of the truncated LcnB and additionally corroborated the PrtP cleavage site in LcnB bacteriocin. In addition, it was demonstrated that, once truncated, LcnB is not able to bind its receptor and is susceptible to additional hydrolysis. This is the first report on proteolytic inactivation of bacteriocins inside the same bacterial host.

## Introduction

Proteolytic system of lactic acid bacteria (LAB) occupied much attention since the 1980s (Law and Kolstad, 1983) onward, given its importance for efficient bacterial growth and the role it plays in industrial processes where these bacteria are found in diverse applications. There is a large amount of data in literature, including some excellent reviews, regarding composition, physiology, biochemistry, diversity and genetics of proteolytic system in different LAB, especially in lactococci (Pritchard and Coolbear, 1993; Law and Haandrikman, 1997; Savijoki et al., 2006). Generally, lactococcal proteolytic system comprises: (a) large cell-envelope proteinase (CEP), responsible for protein digestion and generation of oligopeptides; (b) multiple oligopeptide transporters, ATP-binding cassette transporter proteins that mediate the uptake of proteinase-produced peptides; and (c) intracellular peptidases, enzymes responsible for amino acid release from transported peptides. Historically first discovered and the most studied system is the one present in *Lactococcus lactis*. Application of its strains has long tradition in cheese, butter and other dairy production, branding *L. lactis* as one of the most exploited bacteria overall. Consequently, this makes its CEP PrtP one of industrially most important enzymes. Given that *L. lactis* is auxotrophic for a number of amino acids, PrtP represents essential component of the system providing peptides to the cells and enabling their rapid growth. Bacteriocins are small ribosomally synthesized antimicrobial peptides, produced by some bacteria to antagonize competing ones. Likewise, they have been thoroughly studied, and large literature data is available on broad array of different bacteriocins (Diep and Nes, 2002; Cotter et al., 2005, 2013; Gabrielsen et al., 2014). Given their antimicrobial nature, and the lack of new and efficient antibiotics, bacteriocins are recognized as one of the plausible means for combating the antibiotic-resistant bacteria and their biofilms (Cotter et al., 2013; Mills et al., 2017; Mathur et al., 2018).

In our previous study (Vukotic et al., 2015), we have shown that proteinase PrtP alters the bacteriocin LcnB activity of *L. lactis* subsp. *lactis* BGMN1-501. That discovery was instigated by the analysis of the *lcnB* gene promoter activity, during bacterial growth in media with rising concentrations of casitone. Surprisingly, it was observed that activity of the *lcnB* gene promoter was decreasing while the antimicrobial activity of LcnB was increasing. It was concluded that some other mechanism is compensating for the silencing of the *lcnB* gene transcription. Since the transcription of *prtP* gene is also medium dependent, but in opposing manner, we set to investigate the possible interplay between these two proteins. Namely, the possibility of LcnB being a substrate for PrtP was studied, both by making PrtP^-^ mutants and monitoring their bacteriocin activity, as well as by treating LcnB with proteinase extract and tracking the same trait. It was shown that PrtP is capable of impairing bacteriocin LcnB activity, as its zones of growth inhibition were enlarged in PrtP^-^ mutant and diminished in the PrtP extracts treatment. Nevertheless, whether or not cleavage actually happens *in vivo* and at which position in LcnB remained to be unambiguously evidenced. More importantly, the effect of the hydrolysis on the activity of the single bacteriocin molecule remained open. Hence, the aim of the present study was to confirm proteolytic cleavage of LcnB by PrtP in bacterial culture of BGMN1-501, establish the exact position(s) where the cleavage occurs and determine how this process reflects on the antimicrobial activity of resulting molecules. In addition, as it is known that LcnB binds components of the mannose phosphotransferase system (man-PTS) (Diep et al., 2007), responsible for glucose uptake, we addressed the activity of cleaved bacteriocin molecules in this manner.

## Materials and Methods

### Bacterial Strains, Growth Mediums and Conditions

The bacterial strains and plasmids used in this study are listed in Table 1. *Lactococcus lactis* subsp. *lactis* BGMN1-501 (Kojic et al., 2006) was grown in M17 medium (Oxoid) supplemented with D-glucose (0.5% w/v) (GM17) at 30°C. In addition, chemically defined medium (CDM) was made according to Miladinov et al. (2001). *Escherichia coli* DH5α and ER2523, used for cloning and propagation of constructs, were grown in Luria-Bertani (LB) broth (Miller, 1972) aerobically at 37°C, unless otherwise specified. Agar plates were made by adding 1.5% (w/v) agar (Torlak Belgrade, Serbia) to the liquid media. Ampicillin in concentration 100 μg/mL was used for selection and maintaining of transformants.

**Table 1:** The bacterial strains and plasmids used in the study.

Strains	Relevant characteristic(s)	Source or References
*Lactococcus lactis* subsp. *lactis*		
BGMN1-501	Derivate of *Lactococcus lactis* subsp. *lactis* BGMN1-5 with pMN80 plasmid, LcnB^+^, PrtP^+^, LcnB^r^	Kojic et al., 2006
BGMN1-596	Plasmid free derivate of *Lactococcus lactis* subsp. *lactis* BGMN1-5, LcnB^-^, PrtP^-^, LcnB^s^	Kojic et al., 2006
*Lactococcus lactis* subsp. *cremoris*		
NCDO712	PrtP^+^, Lac^+^	Gasson, 1983
*Escherichia coli*		
DH5α	*aaa^-^ Φ80dlacZΔ*M15 *Δ(lacZYA-argF)U169 recA1 endA1 hsdR17(rk^-^mk^-^) supE44thi-1gyrA relA1*	Hanahan, 1983
ER2523	*fhuA2 [lon] ompT gal sulA11 R(mcr-73::miniTn10–TetS)2 [dcm] R(zgb-210::Tn10–TetS) endA1*Δ*(mcrC-mrr)114::IS10*	New England Biolabs, Ltd., United Kingdom
Plasmids		
pMAL-c5X	pMB1 origin, *lacI*, *malE*, *bla*, Factor Xa cleavage site; 5677 bp	New England Biolabs, Ltd., United Kingdom
pMAL-cX5I	pMAL-c5X carrying part of *lcnB* gene (without leader peptide-encoding DNA sequence) amplified using LcnB-Fw and LcnB-Rev primers by Vent DNA Polymerase	This study
pMAL-cX5II	pMAL-c5X carrying truncated *lcnB* gene amplified using LcnB^∗^-Fw and LcnB-Rev primers by Vent DNA Polymerase	This study
pMN80	80 kbp plasmid carrying *prtP* and *lcnB* genes	Kojic et al., 2006

*LcnB^+^, lactococcin B producer; PrtP^+^, proteinase PrtP producer; LcnB^*r*^, resistance to LcnB; LcnB*^-^*, absence of LcnB production; PrtP*^-^*, proteinase PrtP non-producer; LcnB^*s*^, sensitivity to LcnB; Lac^+^, lactose fermentation ability*.

### Bacteriocin LcnB Purification From *Lactococcus lactis* subsp. *lactis* BGMN1-501

For the purification and mass spectrometric analysis of the bacteriocin in *Lactococcus lactis* subsp. *lactis* BGMN1-501, the strain was grown at 30°C for 16 h in 500 mL of chemically defined growth medium (CDM)with addition of 0.5% casitone (Difco Laboratories, Becton Dickinson and Company, Franklin Lakes, NJ, United States). The bacteriocin was purified following the protocol of Mirkovic et al. (2016). Briefly, the cells were removed by centrifugation (4,500 × *g* for 30 min at 4°C), while the supernatant was saturated up to 40% with ammonium sulfate, and precipitated with stirring for 2 h at 4°C. The precipitate was collected by centrifugation (10,000 × *g* for 30 min at 4°C) and then dissolved in 5 mL of Milli-Q water containing 0.1% trifluoroacetic acid. The bacteriocin was further purified using reverse-phase high-performance liquid chromatography (HPLC). Reverse-phase chromatography of the bacteriocin sample was performed using an Äkta Purifier 10 system (GE Healthcare, Uppsala, Sweden) with a Discovery BIO Wide Pore C_5_column (10 cm by 4.6 mm; particle size, 5 μm; Supelco, Bellefonte, PA, United States). The peptide was eluted using an acetonitrile gradient (0 to 90% with 0.1% trifluoroacetic acid for 10 column volumes). The chromatography was monitored by measuring absorbance at 215 nm. Twenty six obtained fractions were dried and dissolved in 10 μL of Milli-Q water, and all volume was used for antibacterial activity.

### Mass Spectrometry Analysis of Antimicrobial Active Fraction

Mass spectrometry analysis of the active fraction was carried out using a mass spectrometer coupled with HPLC. The sample was injected onto a reverse-phase C18 column (RRHT column; 4.6 by 50 mm; particle size, 1.8 μm) coupled with a Zorbax Eclipse XDB-C18 column installed in a 1200 series HPLC system (Agilent Technologies). The sample components were separated using an acetonitrile gradient (5 to 95% with 0.2% formic acid for 10 min and then 95% for 5 min). The mass spectrometer, a 6210 TOF liquid chromatography-mass spectrometry (LC-MS) system (G1969A; Agilent Technologies, Santa Clara, CA, United States), was run in positive ESI mode with a capillary voltage of 4,000 V, a fragmentor voltage of 200 V, and a mass range of m/z 100 to 3,200. Agilent MassHunter Workstation software and Analyst QS were used for data processing.

### Bacteriocin Activity

Bacteriocin activity of BGMN1-501, as well as of recombinantly produced bacteriocin was evaluated by an agar-well diffusion test (Lozo et al., 2004).

The activity of HPLC-obtained fractions and of chemically synthesized bacteriocin was evaluated by spot-on-lawn assays. Soft GM17 agar (0.75%, w/v) containing sensitive strain was overlaid onto thin GM17 agar plates. After soft agar solidified, 10 μL of sample was spotted and plates were incubated at 30°C overnight. Clear zones of growth inhibition in the spots were used as positive signals. *Lactococcus lactis* subsp. *lactis* BGMN1-596 was used in all tests as sensitive strain.

Proteinase extract was prepared as following: BGMN1-501 was grown on milk citrate agar plates, collected and re-suspended in 0.1 mol/L sodium phosphate buffer (NaPi, pH 7), incubated at room temperature for 30 min, pelleted by centrifugation and supernatants were collected prior to reactions. *In vitro* testing of effect of proteinase extract on bacteriocin activity was done as previously described (Vukotic et al., 2015), except that recombinant bacteriocins were used as substrates in the reactions.

### Bacteriocin Competition Assay

The possibility of truncated peptide LcnB^∗^ to compete with wild type LcnB for binding to its receptor molecule was tested in bacteriocin competition assay. Bacteriocin assay was prepared, as explained above. Before addition of bacteriocin producer, 10 μL of increasing concentrations (62.5–1,000 μg/mL) of LcnB^∗^ was added to the wells made in soft agar and left for 30 min at room temperature to be completely adsorbed. After that, 50 μL of fresh BGMN1-501 culture was added to the wells, and incubated overnight at 30°C to produce growth inhibition zones.

### Glucose-Uptake Inhibition Assay

To test whether truncated peptide can interfere with glucose import, growth of BGMN1-596 in GM17 with added LcnB^∗^ was monitored. Fresh overnight culture, grown in GM17, was inoculated at 5% to fresh media, and saturated with increased concentrations (0–1,000 μg/mL) of chemically synthetized LcnB^∗^. As a control, the same inoculum was made in media without glucose. Bacterial growth was monitored in microtitar plates by OD measurement at 600 nm at different time points for 6h. The assay was done in triplicate.

### DNA Manipulations

Plasmid DNA from lactococci was isolated with the method described by O’Sullivan and Klaenhammer (1993), while QIAprep Spin Miniprep Kit was used for plasmid DNA isolation from *E. coli*, according to the manufacturer’s recommendations (Qiagen, Hilden, Germany). Vent^^®^^DNA Polymerase (New England Biolabs, Ltd., United Kingdom) was used to amplify DNA fragments by PCR, using GeneAmp PCR system 2700 thermal cycler (Applied Biosystems, Foster City, CA, United States). The primers used in PCR for amplification of the *lcnB* gene without leader peptide-encoding DNA sequence and its 18 bp shorter truncated form, the *lcnB^∗^*, are listed in Table 2. The PCR amplified DNA fragments were purified using QIAquick PCR Purification Kit as described by the manufacturer (Qiagen). T4 DNA ligase (Agilent Technologies, United States) was used for DNA ligation of PCR products into pMAL-c5X vector predigested with *Xmn*I, according to the manufacturer’s recommendation. Since VentDNA Polymerase was used, blunt PCR amplicons were obtained, which was suited for ligation with pMAL-c5X vector, opened with *Xmn*I. Since blunt ligation offers two possible orientations, ligation mixtures were transformed in DH5α competent cells, to screen for the appropriate fragment orientations. Standard heat-shock transformation was used for plasmid transfer into *E. coli* DH5α (Hanahan, 1983).

**Table 2:** Sequence of specific primers used in this study.

Primer name	Sequence of primer	Template	Source or References
LcnB-Fw	5′-AGCTTGCAGTATGTTATG-3′	pMN80 plasmid DNA	This study
LcnB^∗^-Fw	5′-AGTGCTGGACCATATACTTGG-3′	pMN80 plasmid DNA	This study
LcnB-Rev	5′-TTAGTGGAATGTTTTTCCCC-3′	pMN80 plasmid DNA	This study

The presence of cloned fragments in pMAL-c5X was determined using *Nco*I/*Sac*I digestion, while the orientation was determined by sequencing using Macrogen Sequencing Service (Macrogen, Netherlands). All digestions with restriction enzymes were conducted according to the supplier’s instructions (Thermo Fisher Scientific).

### Chemical Synthesis of Bacteriocin

LcnB^∗^, the truncated form of bacteriocin LcnB lacking first six amino acids (Table 3), was synthesized by Biomatik Corporation (Cambridge, Canada).

**Table 3:** Amino acid sequences of bacteriocin forms used in this study.

Name	Sequence of peptide	Predicted MW (Da)	Source or References
LcnB	SLQYVMSAGPYTWYKDTRTGKTICKQTIDTASYTFGVMAEGWGKTFH	5324	Kojic et al., 2006
LcnB^∗^	SAGPYTWYKDTRTGKTICKQTIDTASYTFGVMAEGWGKTFH	4603	This study

*First six amino acids that are cleaved by PrtP proteinase are underlined in the wt LcnB bacteriocin*.

### Recombinant LcnB and LcnB^∗^ Overexpression in *E. coli* ER2523 and Purification

Plasmid constructs with appropriate fragment orientation, designated as pMAL-cX5I (carrying complete coding sequence for LcnB) and pMAL-cX5II (truncated for six amino acids at N terminus) were transformed into *E. coli* ER2523 competent cells (New England Biolabs). Transformants were selected on LA Petri dishes containing ampicillin (100 μg/mL) at 37°C. Expression of recombinant peptides was carried out at 22°C by induction with 0.1 mmol/L isopropyl β-D-1-thiogalactopyranoside (IPTG) for 2 h. Purification (cell lysis, affinity chromatography, cleavage of fusion protein with Xa protease) was performed according to manufacturer’s instructions (pMAL Protein Fusion & Purification System; New England Biolabs, Ltd., United Kingdom). Purified recombinant LcnB and LcnB*^∗^* bacteriocins were stored at -20°C in CM buffer (20 mmol/L Tris–HCl pH 7.4, 200 mmol/L NaCl, 1 mmol/L EDTA, 1 mmol/L DTT) containing 50% glycerol.

### SDS-PAGE

Recombinant peptides were analyzed by SDS-PAGE, as previously described (Vukotic et al., 2015).

### HPLC of Recombinant Peptides

Both recombinant peptides and the reaction mixtures of LcnB and PrtP were analyzed using the same reverse-phase chromatography as used for bacteriocin purification (see Section “Bacteriocin LcnB Purification From *Lactococcus lactis* subsp. *lactis* BGMN1-501”).

### Bioinformatic Analysis of LcnB

Bioinformatic analysis of LcnB was carried out using JPred4, the latest version of JPred, one of the most accurate protein secondary structure prediction servers (Drozdetskiy et al., 2015). This neural network-based server was used to predict propensity of each amino acid residue in given sequence to form certain type of secondary structure.

## Results

### Purification and Mass Spectrometric Analysis of Bacteriocin LcnB

**Figure 1:**
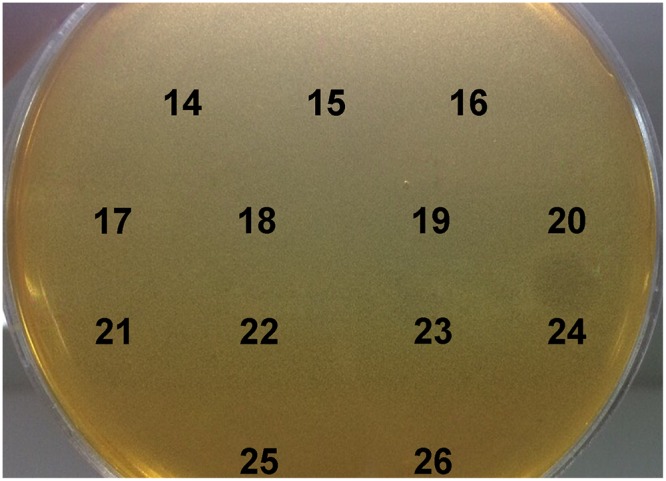
Testing of HPLC-purified fractions of MN1-501 proteins from cell free supernatant for bacteriocin activity. *L. lactis* subsp. *lactis* BGMN1-596 was used as sensitive strain.

**Figure 2:**
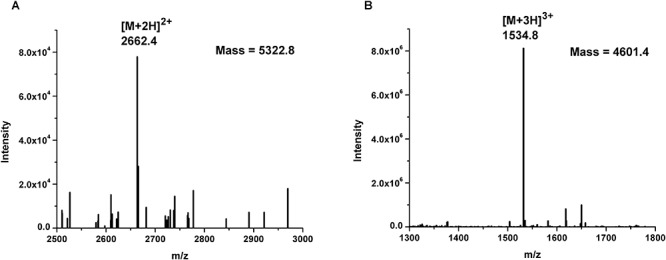
Mass spectrometry results of: **(A)** LcnB and **(B)** truncated peptide (LcnB^∗^) present in HPLC-derived active fraction 24.

BGMN1-501 was successfully cultivated in chemically defined medium and tested positively for bacteriocin activity (data not shown). The proteins present in the supernatant were precipitated by ammonium sulfate precipitation at 40% saturation, dialyzed and separated by RP-HPLC. Twenty-six obtained fractions were concentrated and tested for antimicrobial activity using spot-on-lawn method. The active fraction (fraction 24, Figure 1) was analyzed by ESI-TOF MS and the molecular mass of the present peptides was determined. Beside the [M + 2H]^2+^peak corresponding to predicted molecular mass of wild type LcnB/ (Figure 2A), additional peptide was detected (Figure 2B). The mass analysis showed that the recorded molecular ion of this peptide was *m*/*z* 1534.8 ([M + 3H]^3+^), which indicated the molecular mass of 4601.4 Da. Based on LcnB amino acid sequence analysis, the detected peptide corresponded perfectly to theoretical LcnB truncated of the first six amino acids of the mature peptide.

### Truncated Form of LcnB (LcnB^∗^) Is Not Active

When the presence of two peptides in the active fraction was established, we set out to determine and possibly compare the activity of both. We ordered chemically synthesized truncated form of LcnB, shortened for six amino acids from N terminus, from Biomatik (Cambridge, Canada) and found it inactive on sensitive strain BGMN1-596, even in very high concentrations such as 1 mg/mL. However, since the manufacturer could not produce the wild type peptide, we decided to produce both peptides by means of genetic engineering. LcnB and LcnB^∗^ were produced as their mature peptide forms using pMAL protein fusion and expression system. Briefly, using plasmid pMN80 as template, both *lcnB* without leader peptide-encoding DNA sequence and its 18 bp shorter form were amplified with PCR and cloned in pMAL-c5X to be in-frame with maltose binding protein (MBP), and transformed into ER2523 strain for overexpression. The transformants were selected and propagated at 22°C for overexpression and purification, because the induction of expression under normal conditions (37°C) resulted in insoluble proteins of interest. Similar results were also observed by other researchers (Chung et al., 2011; Miljkovic et al., 2018). Overview of the cloning and expression process is given in Supplementary Figure S1.

**Figure 3:**
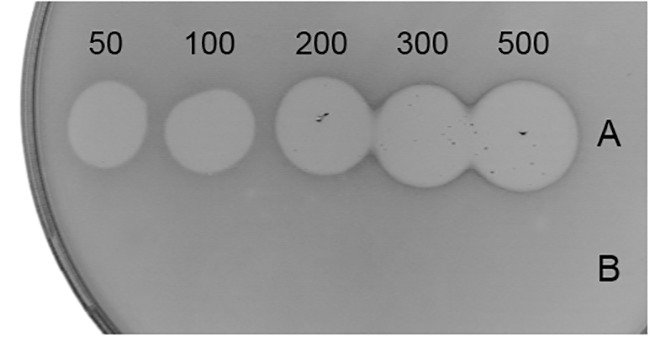
Antimicrobial activity of recombinant LcnB **(A)** and LcnB^∗^ **(B)**. Growing concentrations (50–500 μg/mL) of each bacteriocin were spotted on lawn of sensitive BGMN1-596 cells. LcnB activity is concentration dependent while LcnB^∗^ does not possess antimicrobial activity.

When tested in spot-on-lawn bacteriocin activity assay, LcnB demonstrated strong antimicrobial activity, dependent on applied concentration. However, LcnB^∗^ proved to be inactive regardless of the concentration applied (Figure 3).

### *In vitro* PrtP Digestion of Recombinant LcnB Yields LcnB^∗^

**Figure 4:**
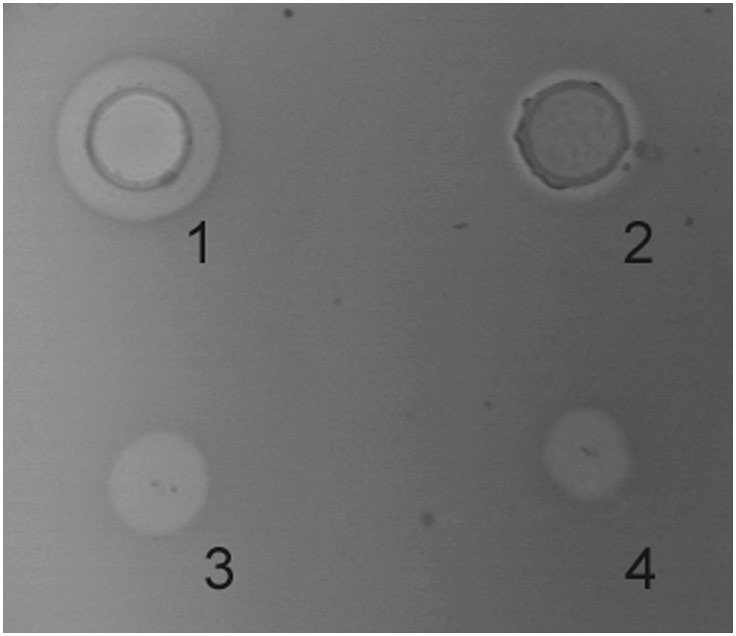
Antimicrobial activity of recombinant LcnB before and after 3 h incubation with PrtP proteinase in agar-well diffusion (1–2), and in spot-on-lawn (3–4) assays.

In order to confirm the premises put forward by previous results, recombinant LcnB was subjected to PrtP hydrolysis *in vitro* and monitored for activity and peptide generation. As expected, PrtP extract reduced the bacteriocin activity of LcnB after 3 h of incubation as zones of growth inhibition diminished, both in spot-on-lawn and agar-well test (Figure 4). However, some residual activity remained, which had also been demonstrated in our previous work on wild type bacteriocin, isolated from BGMN1-501. After 20 h of incubation with proteinase PrtP, no activity could be detected (data not shown).

**Figure 5:**
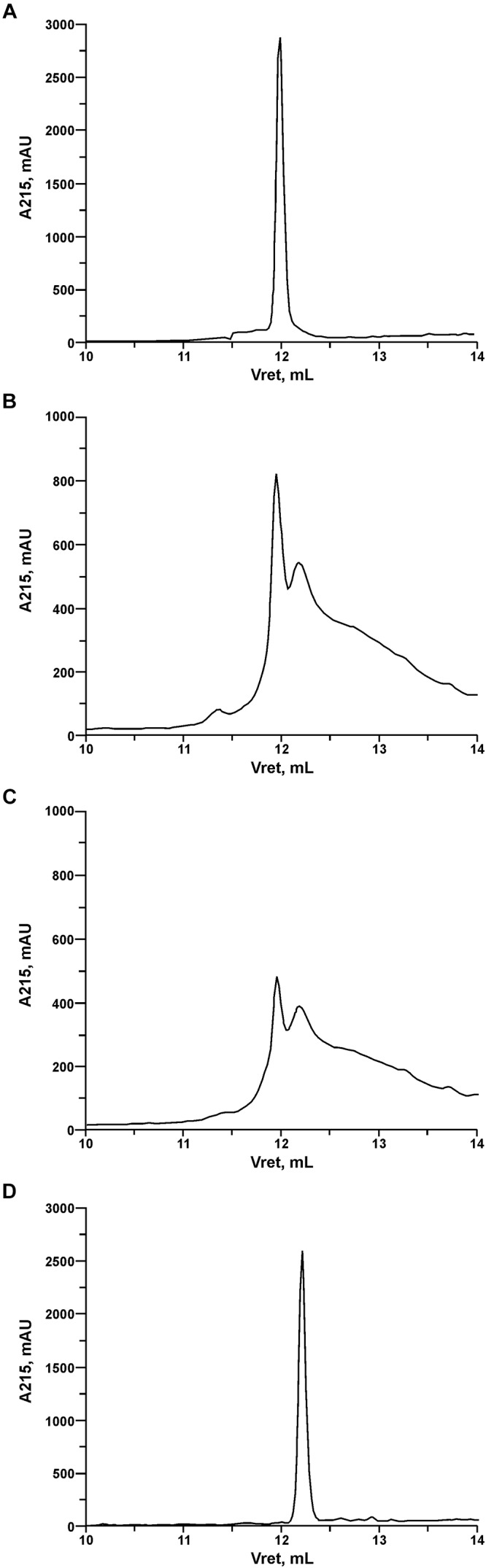
RP-HPLC of recombinant LcnB **(A)**, recombinant LcnB hydrolyzed with PrtP for 3 h **(B)**, recombinant LcnB hydrolyzed with PrtP for 20 h **(C)** and recombinant LcnB^∗^ **(D)**. Details are given in the text.

In order to investigate the pattern and kinetics of PrtP hydrolysis of LcnB, the components of digestion mixture were separated using reverse-phase HPLC after 3 and 20 h of digestion. In addition, recombinant LcnB as starting substrate and LcnB^∗^ as potential product were also loaded on the same column and separated under the same conditions, to establish the adequate controls (Figure 5). As can be seen on the figure, retention volumes of controls in given conditions were 11.9 mL for wt LcnB (Figure 5A), and 12.2 mL for LcnB^∗^ (Figure 5D). As for the 3 h hydrolysis (B), it can be seen that another peak emerged in the close vicinity of the original one, perfectly corresponding to the peak generated by LcnB^∗^. In addition, further degradation can be noted in the background. With the prolonged time of hydrolysis (Figure 5C), it is clear that the amount of original substrate LcnB is drastically reduced, however, the same is happening to the LcnB^∗^ peptide – suggesting that apart from the specific digestion occurring at sixth amino acid in the peptide, hydrolysis extends to other peptide bonds forming a background signal, notable on the chromatogram.

### LcnB^∗^ Does Not Interact With LcnB Receptor

The presence of LcnB^∗^ in the highest concentration did not alter bacterial growth, given that bacteria grew evenly well in the presence as in the absence of LcnB^∗^ (Supplementary Figure S2). In addition, pretreatment of sensitive MN1-596 cells with high concentrations of LcnB^∗^ did not influence the latter bacteriocin activity of wild type molecule on the same cells (Supplementary Figure S3). Taken together, these results testify that truncated molecule does not bind nor block the receptor for LcnB on the surface of sensitive cells.

### Bioinformatic Analysis Predicts Loss of Secondary Structure in LcnB^∗^

**Figure 6:**
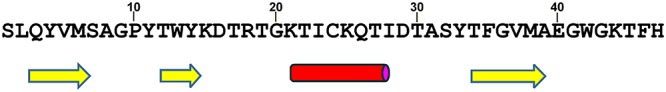
JPred4 prediction of secondary structure formation in LcnB. Yellow arrows: β-strands; Red cylinder: α-helix.

JPred4 analysis of amino acid sequence of wild type bacteriocin predicted three β-strands and one α-helix (Figure 6). The first β-strand comprises four residues from the first six amino acids (QYVM) of the peptide, implying that this part of the peptide has an important function in secondary structure formation.

## Discussion

In our previous paper, we described the medium dependent regulation of bacteriocin LcnB activity in BGMN1-501, and gave the first insights into interaction between proteinase PrtP and LcnB bacteriocin. Through the generation and analyses of single-gene knockouts it was shown that bacteriocin activity is negatively regulated by PrtP, which is on the other hand negatively regulated by the peptide concentration in the growth medium. Finally, it was demonstrated that PrtP impaired LcnB activity *in vitro*. However, an *in vivo* study was needed to confirm whether this unusual hydrolysis actually occurs and consequently to determine at which peptide bond, as well as its severity to the function of bacteriocin. This was also interesting from the point of bacteriocin mode of action, given that structure and receptor-biding domain are still undefined for this bacteriocin.

To approach this problem, we decided to purify and detect the bacteriocin present in the overnight culture of BGMN1-501, using mass spectrometry. The rationale for this was the following: if the hydrolysis of LcnB does not occur, a peak of adequate bacteriocin mass would be detected in the active fraction; if, however, the hydrolysis of LcnB indeed occurs, we would either detect one peptide of smaller mass or two peptides of different masses inside the active fraction, or two active fractions each carrying an active peptide.

To determine the form(s) of LcnB present in BGMN1-501 culture, the strain was grown in chemically defined minimal medium, supplemented with 0.5% casitone. This was done for two reasons: (i) to grow bacteria in conditions favoring PrtP expression and hence putative bacteriocin digestion, and (ii) to reduce the presence and number of molecules which could interfere with subsequent manipulations, i.e., chromatographic separation and detection of molecules of interest. Mass spectrometry revealed two peptides present in active fraction, one corresponding to theoretical bacteriocin LcnB, and the other of smaller mass. The peptide sequence analysis revealed that the obtained 721 Da difference between the two detected peptides corresponded perfectly to theoretical mass of first six amino acids in LcnB. This strongly indicated that the additional peptide present in the active fraction is indeed the truncated form of LcnB.

This finding confirmed that LcnB actually gets hydrolyzed during bacterial growth. However, since both peptides were detected in the active fraction, the activity of the truncated form, named LcnB^∗^, could not be determined. To define and compare the activity of both forms of bacteriocin, they were produced with cloning work using pMAL expression system. Once purified, both peptides were tested for antimicrobial activity, but growth inhibition zone was produced only by wild type peptide LcnB, while LcnB^∗^ expressed no activity. This was also the case when chemically synthesized truncated bacteriocin was applied.

Chromatographic analysis of pattern and kinetics of PrtP-LcnB digestion, paralleled with bacteriocin activity assays, gave the final confirmation of our work. The results demonstrated that once exposed to PrtP, LcnB gets converted to LcnB^∗^ and loses activity. The cleavage site was confirmed to be between sixth and seventh amino acid in the peptide, as the retention volume of the peak generated in hydrolysis matched the peak generated by recombinant LcnB^∗^. It also became evident that additional hydrolysis occurs with time. Therefore, it can be concluded that the preferred site for PrtP action is methionine-serine bond at the N terminus of LcnB, however, peptides liberated by this action may be further hydrolyzed with time.

Finally, the deleterious effect of the missing amino acids on structuring the bacteriocin molecule was ascertained with bioinformatic analysis of LcnB. Given that detailed 3D structure of this bacteriocins is yet undetermined, the influence of given six amino acids removal on the final protein structure is hard to predict. However, secondary structure prediction suggested that these six amino acids are involved in β-sheet formation. Given that this β-sheet is predicted to be rather small, consisting of three strands and only 12 amino acids, it is highly probable that deletion of first β-strand, induced by PrtP, destabilizes the structure completely, leading to denaturation of this β-sheet.

The loss of antimicrobial activity after here described deletion by PrtP proteinase, suggests that LcnB receptor binding domain is functionally disrupted without first six amino acids and cannot interact with the receptor molecule. This conclusion is supported with results obtained in glucose-uptake inhibition experiment where it was demonstrated that bacterial growth is not altered in the presence of LcnB^∗^ meaning that truncated peptide does not interfere with glucose import by man-PTS, which is bacteriocin target molecule. Likewise, bacteriocin competition assay confirmed the lack of competition between LcnB^∗^ and LcnB for the receptor. The experiment was set according to Ovchinnikov et al. (2014) who determined the receptor binding domain of another bacteriocin, LsbB, by producing an array of different bacteriocin-derived peptides and tracking the blocking activity of each peptide in microtitar plate assay. We used the same logic in bacteriocin competition assay, in which we pre-incubated sensitive cells with LcnB^∗^ to allow its attachment to the receptor molecules, and then performed standard bacteriocin test on the same cells. It turned out that LcnB exhibited antimicrobial effect regardless of the presence of even high concentration of LcnB^∗^. This result demonstrated that LcnB^∗^ is not able to compete with full-length peptide for the receptor, or in other words, does not bind components of man-PTS at all.

Taking all the results in consideration, we can conclude that our newest findings confirm several hypotheses formed in our previous work. Proteinase PrtP indeed hydrolyzes bacteriocin LcnB *in vivo*, but by doing so, inactivates it completely through destruction of its receptor binding domain. The reason for this, however, eludes the common microbial logic. There seems to make no sense for bacteria to destroy their own weapons for competition. However, this risk appears merely when PrtP is excessively expressed, which happens only when bacteria grow in nitrogen-depleted media, and are in other words starving. Nevertheless, it should be emphasized that conditions applied in this study mimicked such situations, and that active bacteriocin was present in bacterial culture. Hence, this “self-digestion” only partially jeopardizes host antimicrobial potential, as it seems that LcnB degradation by PrtP is never so intense to majorly deplete bacteria of their antimicrobial potential. This is why we consider that there is physiological explanation for this process. The fate of generated peptides may be various; for example they may be involved in signaling or serve as a source of amino acids for the starving bacterial cells, etc. These are challenging presumptions, and future research will be driven to address this issue.

## Author Contributions

GV, NP, NM, and NS contributed to acquisition and analysis of data. GV, BJ, MK, and DF contributed to design of the work, analysis, and interpretation of the data. GV, NP, and BJ drafted the manuscript. MK contributed to the conception of the work and critically revised the final version to be published.

## Conflict of Interest Statement

The authors declare that the research was conducted in the absence of any commercial or financial relationships that could be construed as a potential conflict of interest.

## Supplementary Material

FIGURE S1 Production of LcnB and LcnB^∗^ by recombinant DNA technology. **(A)** PCR amplification of *lcnB* (1, 2) and its truncated form (3, 4); 5, Generuler DNA Ladder Mix. **(B)** *Nco*I/*Sac*I digestion of cloned PCR products in pMAL-c5X: 1–2, construct pMAL-c5XI (carrying *lcnB*); 5–6, construct pMAL-c5XII (carrying truncated form of *lcnB*); 3 and 7, empty vector; 4, Generuler DNA Ladder Mix. Note – the 90 bp increases in size compared to PCR amplicons is derived from vector. **(C)** SDS-PAGE of overexpressed clones in *E. coli* ER2532: 1 and 3, fusions with MBP; 2 and 4, arrows pointing to proteins of interest (LcnB and LcnB^∗^, respectively) released from MBP by Xa protease; 5 spectra multicolor broad range ladder.

FIGURE S1 Glucose-uptake inhibition assay. Growth of BGMN1-596 was monitored in GM17 enriched with different concentrations of LcnB^∗^. Each line represents growth with corresponding concentration of LcnB^∗^, noted in the picture. Bacteria were also grown in absence of glucose (M17). Bacterial inoculum was 5% from fresh overnight culture.

FIGURE S3 Bacteriocin competition assay. Antimicrobial activity of BGMN1-501 was tested on sensitive cells pretreated 30 min with different concentrations of LcnB^∗^: (1) negative control BGMN1-596; (2–6) pretreatment done with 1,000; 500; 250; 125, and 62.5 μg/mL of LcnB^∗^, respectively, (7) pretreatment done with 10 mmol/L NaPi buffer (pH 7) used to dissolve LcnB^∗^.
