# Supplementary figures and images for: Lactococcin B Is Inactivated by Intrinsic Proteinase PrtP Digestion in Lactococcus lactis subsp. lactis BGMN1-501
Source: Front Microbiol. 2019 Apr 24;10:874. doi: 10.3389/fmicb.2019.00874 (PMC6491934; doi:10.3389/fmicb.2019.00874)

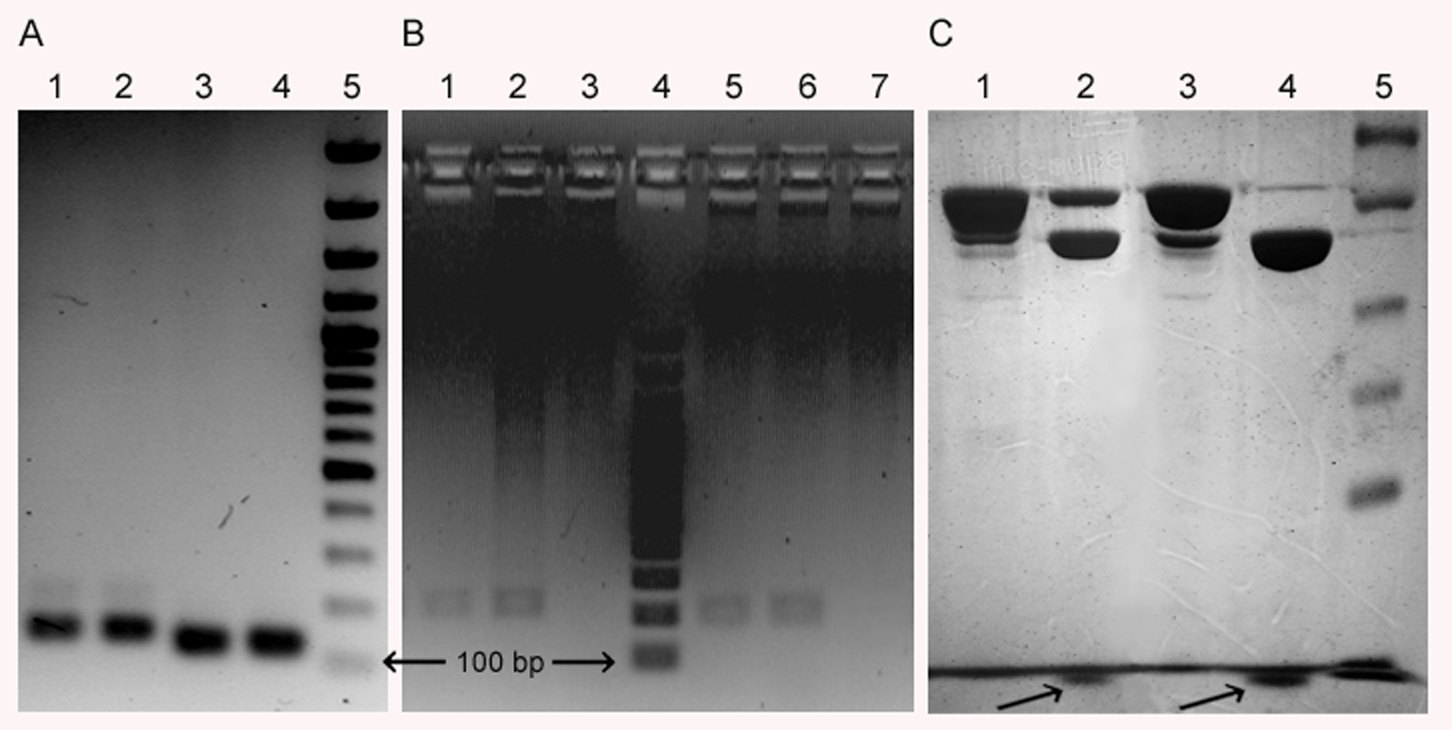

Supplement: FIGURE S1 — Production of LcnB and LcnB∗ by recombinant DNA technology. (A) PCR amplification of lcnB (1, 2) and its truncated form (3, 4); 5, Generuler DNA Ladder Mix. (B) NcoI/SacI digestion of cloned PCR products in pMAL-c5X: 1–2, construct pMAL-c5XI (carrying lcnB); 5–6, construct pMAL-c5XII (carrying truncated form of lcnB); 3 and 7, empty vector; 4, Generuler DNA Ladder Mix. Note – the 90 bp increases in size compared to PCR amplicons is derived from vector. (C) SDS-PAGE of overexpressed clones in E. coli ER2532: 1 and 3, fusions with MBP; 2 and 4, arrows pointing to proteins of interest (LcnB and LcnB∗, respectively) released from MBP by Xa protease; 5 spectra multicolor broad range ladder. [file Image_1.TIF]

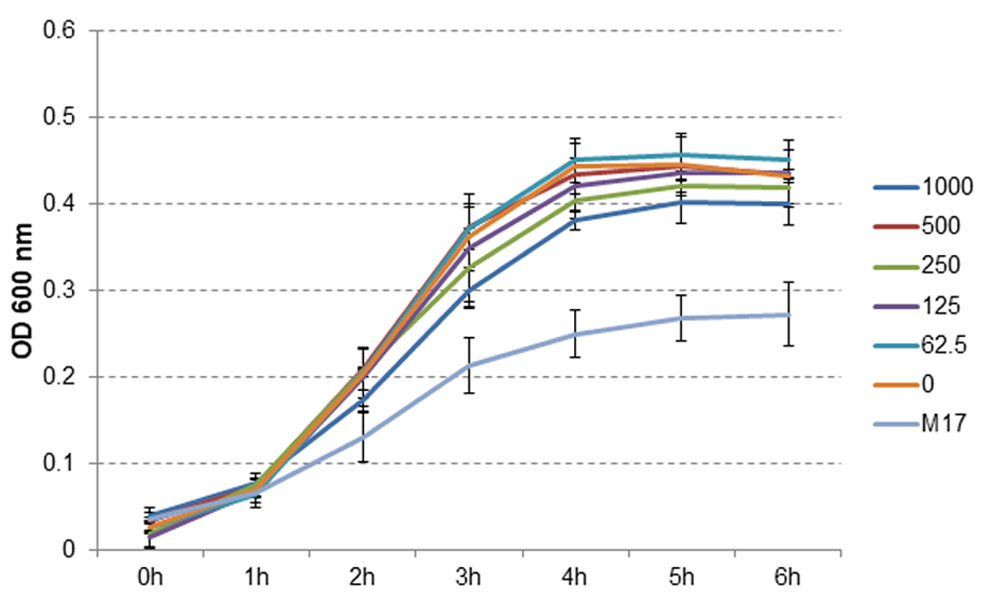

Supplement: FIGURE S1 — Glucose-uptake inhibition assay. Growth of BGMN1-596 was monitored in GM17 enriched with different concentrations of LcnB∗. Each line represents growth with corresponding concentration of LcnB∗, noted in the picture. Bacteria were also grown in absence of glucose (M17). Bacterial inoculum was 5% from fresh overnight culture. [file Image_2.TIF]

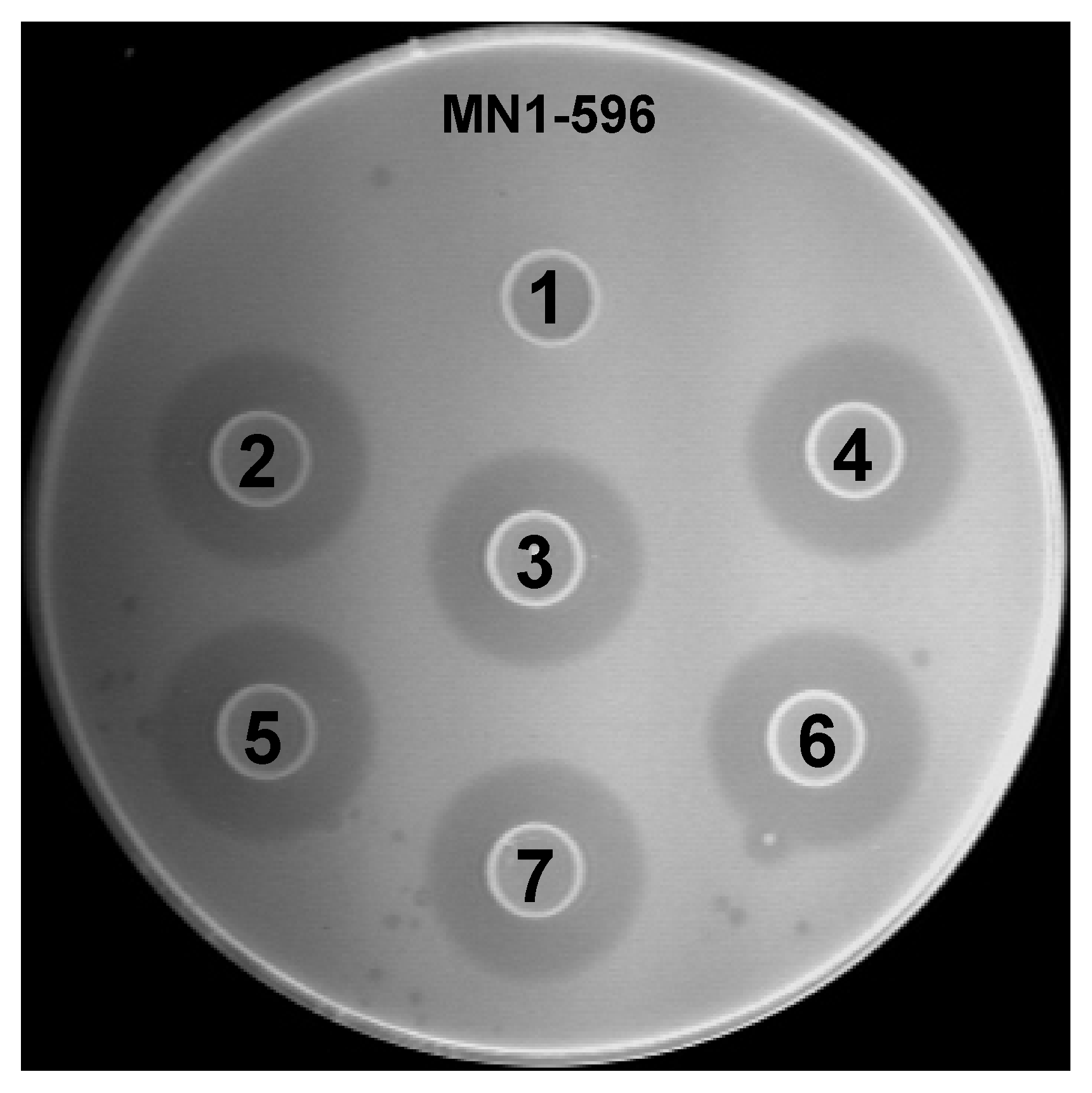

Supplement: FIGURE S3 — Bacteriocin competition assay. Antimicrobial activity of BGMN1-501 was tested on sensitive cells pretreated 30 min with different concentrations of LcnB∗: (1) negative control BGMN1-596; (2–6) pretreatment done with 1,000; 500; 250; 125, and 62.5 μg/mL of LcnB∗, respectively, (7) pretreatment done with 10 mmol/L NaPi buffer (pH 7) used to dissolve LcnB∗. [file Image_3.TIF]
